# Gastric Surgery in Institutional Treatment

**Published:** 1913-03-22

**Authors:** 


### NEW DEVELOPMENTS IN MEDICINE.

### II.—Gastric Surgery in Institutional Treatment.

When physiology, mainly through the observations of Beaumont on the trapper Alexis St. Martin, had elucidated and clarified its conception regarding the functions of the stomach, surgery was as yet impotent to deal with many of the most serious affections of that organ. The writings of older physicians and surgeons display a wide and thorough clinical knowledge of these diseases, amplified by an acquaintance with pathological detail which, considering the difficulties under which that knowledge was amassed, is striking enough. But at the same time they give little indication of a tendency to adopt what to-day would be called rational lines of treatment. Nor was it possible to attack the stomach at a time when an abdominal section meant the probable death of the patient. The introduction of anæsthetics, rendering possible the performance of operations on a quiescent patient, and the era of aseptic surgery initiated by Semmelweiss and Lister, made it necessary for surgeons to reconsider their old views. The "indications" for surgical interference in stomach diseases were revised. They are being revised to-day, for we have progressed considerably during the last decade so far as this branch of the surgeon's art is concerned. To give a clear idea of the extent and importance of such revision means to write a chapter of the history of surgery, one of the most fascinating perhaps of all its chapters. But these articles are limited solely to the relations between new developments and institutional interests.

In how far, then, have these new revisions in gastric surgery influenced institutional treatment? To broaden the inquiry, in how much has such progress in surgical treatment of stomach diseases modified certain conceptions entertained by institutional workers? The true answer to this question is not an easy one. The advance along this line has been synchronous with advance in other departments, and chiefly in that of general surgery, of which gastric surgery is, after all, merely a special branch. This much may clearly be admitted, that but for the marked development in abdominal surgery there would not to-day be that tendency to elaboration on the surgical side which undoubtedly exists. A major operation on the stomach demands for its successful performance the possession on the part of the operator of the highest surgical skill and of the most perfect asepsis of a technically educated staff of assistants and of trained and efficient nurses. These major operations, as undertaken in every large hospital to-day, are, some of them at least, highly complicated in technique. The whole stomach has repeatedly been excised in cases of cancer; its entire middle has been removed by plastic operations of the type of circular gastrectomy; it has been opened through the abdominal wall so as to form a permanent passage for food (gastrotomy) in a starving patient with an obstruction in the œsophagus; it has been anastomosed in several methods to the surrounding and underlying gut; it has been folded on itself and its natural openings have been enlarged (gastroplication and gastroraphy); it has been removed in sections for ulcers and new growths; and it has been moored into various positions (gastropexy). Nor does the list end here, for during the past five years an entirely new branch of stomach work has been developed by the researches of Holtzknecht and Cannon. This is the investigation of the stomach by means of the *x*-rays. Nor must it be forgotten that with the development of the instruments for direct illumination, which made it possible to see into the hollow viscera, such as the trachea, œsophagus, and bladder, the desire to perfect such instruments for stomach work was not wanting. Nowadays we have at hand the gastroscope, an instrument which permits of direct visual examination of a stomach on an unanæsthetised patient. This instrument, although not much used as yet in this country, has proved its usefulness in Continental hospitals and may now be taken to have justified its adoption.

All this elaboration and development in this special branch of general surgery have conduced to bring about two results. One is the refinement in surgical nursing that is seen in the best hospitals; an "abdominal case," and more especially a grave "stomach case," needs in its successful after-treatment the most skilful nursing. The old type of nurse, uninstructed in dietetics and in the niceties of surgical nursing, could hardly be expected, or trusted, to look after a case where the slightest inattention might result in grave disaster. Special contrivances in aid of the nurse have been evolved and perfected; of these we may mention infusion apparatus and contrivances for enabling the patient to take up special attitudes in bed. The greatest care and skill is required in handling a patient who has just submitted to an operation the success of which depends mainly on the safe holding together of delicate serous and mucous membranes by skilfully adapted layers of stitches. Such gastric cases have undoubtedly tended to give hospital nurses more work, and more exacting work, than they had formerly to deal with. The experience derived from such nursing has been of incalculable value.

In the second place, the progress of gastric surgery has conduced to the perfection of that ideal asepsis in theatre and ward which we now know is absolutely necessary in an institution that claims to be modern and up to date. Where abdominal surgery is a feature of the work done in an institution we generally find that the surgical department of that institution may honestly claim to be above reproach. It is quite true that much excellent work is done in hospitals where the conditions are by no means ideal, but the excellence of the results so obtained is a tribute largely to the high standard of surgical nursing and to the extreme care of the operators for which the institution, in the majority of cases, can scarcely get the credit. It is the duty of every general surgeon attached to a hospital, which admits patients on whom stomach operations may have to be carried out, to insist that the committee shall give him proper facilities for carrying on his work under conditions which are as close to the ideal as it is possible to get them. If such conditions cannot be granted then these cases should not be admitted for both patient and surgeon run a risk which is negligible only in cases of extreme urgency. Ideal conditions do not ordinarily mean extravagance and elaboration. The present tendency is no longer to build and equip sumptuously constructed operating theatres which are supposedly the perfection of aseptic environment. In practice it has been found that such a supposition is a delusion. Simplicity is the modern ideal, not elaboration, but it must be a perfect type of simplicity in which due consideration is paid to every factor for good or evil. On such perfection the surgeon should insist, and he should demonstrate to committee or superintendent that an under-estimation of the risk which is run in neglecting to secure perfection entails difficulties and dangers which ought not to be encountered.

